# Factors associated with a high or low implantation of self-expanding devices in TAVR

**DOI:** 10.1007/s00392-021-01901-3

**Published:** 2021-06-24

**Authors:** Verena Veulemans, Oliver Maier, Kerstin Piayda, Kira Lisanne Berning, Stephan Binnebößel, Amin Polzin, Shazia Afzal, Lisa Dannenberg, Patrick Horn, Christian Jung, Ralf Westenfeld, Malte Kelm, Tobias Zeus

**Affiliations:** 1 Division of Cardiology, Pulmonology and Vascular Medicine, Medical Faculty, Heinrich Heine University, Moorenstr. 5, 40225 Düsseldorf, Germany; 2 CARID (Cardiovascular Research Institute Düsseldorf), Moorenstr. 5, 40225 Düsseldorf, Germany

**Keywords:** TAVR, TAVI, Implantation depth

## Abstract

**Objectives:**

Optimizing valve implantation depth (ID) plays a crucial role in minimizing conduction disturbances and achieving optimal functional integrity. Until now, the impact of intraprocedural fast (FP) or rapid ventricular pacing (RP) on the implantation depth has not been investigated. Therefore, we aimed to (1) evaluate the impact of different pacing maneuvers on ID, and (2) identify the independent predictors of deep ID.

**Methods:**

473 TAVR patients with newer-generation self-expanding devices were retrospectively enrolled and one-to-one propensity-score-matching was performed, resulting in a matching of 189 FP and RP patients in each cohort. The final ID was analyzed, and the underlying functional, anatomical, and procedural conditions were evaluated by univariate and multivariate analysis.

**Results:**

The highest ID was reached under RP in severe aortic valve calcification and valve size 26 mm. Multivariate analysis identified left ventricular outflow (LVOT) calcification [OR 0.50 (0.31–0.81) *p *= 0.005*], a “flare” aortic root [OR 0.42 (0.25–0.71), *p *= 0.001*], and RP (OR 0.49 [0.30–0.79], *p *= 0.004*) as independent highly preventable predictors of a deep ID. In a model of protective factors, ID was significantly reduced with the number of protective criteria (0–2 criteria: − 5.7 mm ± 2.6 vs. 3–4 criteria − 4.3 mm ± 2.0; *p *< 0.0001*).

**Conclusion:**

Data from this retrospective analysis indicate that RP is an independent predictor to reach a higher implantation depth using self-expanding devices. Randomized studies should prove for validation compared to fast and non-pacing maneuvers during valve delivery and their impact on implantation depth.

**Trail registration:**

Clinical Trial registration: NCT01805739.

**Graphic abstract:**

Study design: Evaluation of the impact of different pacing maneuvers (fast ventricular pacing—FP vs. rapid ventricular pacing—RP) on implantation depth (ID). After one-to-one-propensity-score-matching, independent protective and risk factors for a very deep ID beneath 6 mm toward the LVOT (< − 6 mm) were identified. Stent frame pictures as a courtesy by Medtronic^®^. *AVC* aortic valve calcification.

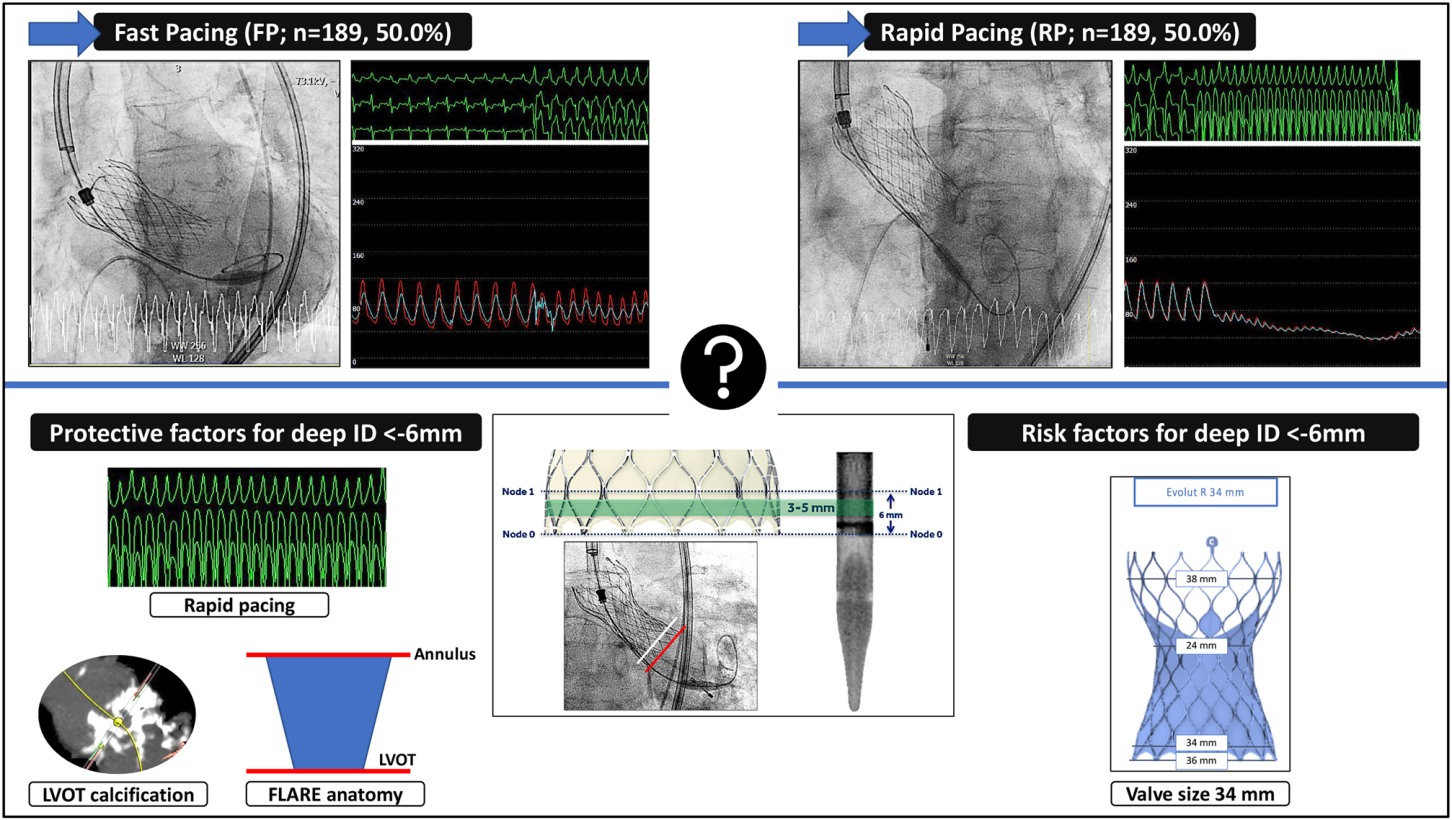

**Supplementary Information:**

The online version contains supplementary material available at 10.1007/s00392-021-01901-3.

## Introduction

Transcatheter aortic valve replacement (TAVR) has been established as the treatment of choice in high-risk patients with severe symptomatic aortic stenosis and has convincing benefits in intermediate- and low-risk patients [1]. Technological improvements such as retrievable valves, smaller sheath sizes, and new skirts have led to outcome optimization [2]. However, postprocedural conduction disturbances following TAVR with self-expanding devices occur frequently, in the range of 15–35% of cases [3, 4]. Optimizing the valve implantation depth (ID) plays a crucial role in avoidance of conduction disturbances and subsequently the need for permanent pacemaker implantation (PPI), and to achieve the best functional integrity [5–7]. Fast (FP) and rapid ventricular pacing (RP) maneuvers are used to temporarily reduce cardiac output, thus allowing for stable and optimal valve deployment. Current data only address hemodynamic side effects and the clinical impact of the TAVR-associated rapid pacing maneuver [8–10]. However, structured data concerning the impact of FP or RP on implantation depth are lacking.

In this study, we aimed to (1) evaluate the impact of different pacing maneuvers on implantation depth in a large cohort of all-comer TAVR patients, and (2) address independent predictors of deep implantation depth.

## Methods

### Study population

From among 1767 consecutive patients with symptomatic severe aortic stenosis (AS) who underwent transfemoral (TF) TAVR with newer-generation self-expanding devices from 2015 to April 2020 at the Heart Centre Düsseldorf, we included 473 patients with complete data in this retrospective analysis. Balloon-expandable valves, valve-in-valve procedures, pure aortic regurgitation, bicuspid valves, TAVR with other self-expanding devices at low count, and procedures without available MSCT data were excluded. Hence, only TF TAVR with the self-expanding CoreValve System (Medtronic Inc., Minneapolis, MN; Evolut R/Pro) was included. All procedures were performed according to current guideline recommendations and under local anesthesia. The initial study cohort was further separated into patients undergoing TAVR under FP (*n* = 284; 60.0%) or RP (*n* = 189; 40.0%). Due to the heterogeneity of the two populations, a propensity-score matching was employed to match RP and FP patients for risk (logistic EuroSCORE I), aortic valve area, aortic valve and left ventricular outflow calcification. One-to-one propensity-score-matching created 189 patients in the FP group and 189 patients in the RP group to a total of 378 patients of the final study cohort. A full overview of the study design and the most important read-outs are displayed in the Graphical abstract.

### Study endpoints

The primary study endpoint was defined as the ID depending on the pacing strategy. Furthermore, we aimed to establish independent predictors for ID.

Secondary endpoints were defined as the impact of RP to FP on thirty-day outcomes according to the VARC-2 definitions [11].

### Procedural details and 3D image analysis of MSCT

Fast pacing (FP) was defined as an episode of ventricular pacing between 100 and 160 bpm to reach a systolic blood pressure less than 100 mmHg during the final valve release. Rapid pacing (RP) was defined as an episode of ventricular pacing between 180 and 200 bpm with the goal of inhibiting cardiac output during the final valve release. Patients without any pacing maneuver were excluded from the analysis due to sample size and power concerns. Fast- and rapid pacing were realized through a temporary pacemaker device using a transfemoral approach.

The final depth of device implantation was calculated in a projection towards the native aortic annulus leaflets (noncoronary cusp—NCC; left coronary cusp—LCC) and a coaxial stent frame position. In detail, the final ID was measured from the edge of the frame up to the nadir of the NCC and LCC. Valve oversizing was calculated as (prosthesis size − native annulus size/native annulus size) × 100.

MSCT images were transferred to a dedicated workstation for 3-dimensional (3D) volume-rendered reconstruction (3 mensio Structural Heart^™^, Pie Medical Imaging BV, Maastricht, The Netherlands) after the procedure according to TAVR-related standardized recommendations for CT image acquisition [12]. Dimensions were determined with the use of workstation tools. A tubular configuration of the aortic root (“tube”) was considered when the mean aortic annulus and LVOT diameter matched in size towards a ratio of 0.95–1.05. A flared configuration was considered when the mean LVOT diameter was smaller than the mean annulus diameter (ratio > 1.05). A tapered configuration (mean diameter of the LVOT greater than the mean annulus diameter) fulfilled the ratio < 0.95. The severity of aortic valve calcification (AVC) was quantified in a semiquantitative manner, resulting in grading from mild to severe.

### Statistical analysis

The collected data included patient characteristics, imaging findings, periprocedural in-hospital data, laboratory results, and follow-up data. Continuous data are described by the mean and standard deviation, and categorical variables by frequencies and percentages. Continuous variables were compared using Student’s *t* test, and categorical variables were compared using Fisher’s exact test.

One-to-one propensity-score-matching was realized according to existent statistical guidelines based on the heterogeneity of the over-all population of the study. Binomial multivariate regression was performed to assess independent predictors of deep ID <  − 6 mm according to the current recommendations. Covariates associated with very deep ID <  − 6 mm in the univariate analysis (*p *< 0.1) were entered into the multivariate model. Independent predictors were built up to a summarized calculation to predict the deep ID. Receiver operating characteristic (ROC) analysis and the c-index (area under the curve, AUC) were used to validate the model fit.

The data analysis was performed using the statistical software SPSS (version 27.0.1, SPSS Inc., Chicago, IL, USA), GraphPad Prism (version 6.0, GraphPad Software, San Diego, CA, USA), and Wizard 2-Statistics and Analysis (Evan Miller). All statistical tests were 2-tailed, and a value of *p *< 0.05 was considered statistically significant.

## Results

### Baseline characteristics

Baseline characteristics did differ according to the particular risk profile and the approach used (FP or RP). Patients undergoing TAVR under RP were predominantly males, had a lower logistic EuroScore I (FP vs. RP: 25.4 ± 14.9 vs. 21.6 ± 14.0; *p *= 0.004*), and a less narrow aortic valve area (FP vs. RP: 0.73 ± 0.2 vs. 0.77 ± 0.2; *p *= 0.012*). In particular, the severity of aortic valve calcification (AVC) and left-ventricular outflow (LVOT) calcification differed between groups with regard to the number of mildly and severely calcified aortic valves. A full overview of the baseline clinical and functional characteristics is displayed in Supplementary material—Table 1.

One-to-one propensity-score matching created 378 patients (FP = 189 and RP = 189). The two propensity-matched groups were more balanced according to their baseline characteristics but did still differ in their risk profiles and in AVC distribution. A full overview of the baseline clinical and functional characteristics is displayed in Supplementary material—Table 2. All further analyses were established in the propensity matched cohorts.

### General procedural characteristics

Procedural details and clinical outcomes are displayed in Supplementary material—Table 1. Contrast use (FP vs. RP: 107.6 ml ± 53.3 vs 86.6 ml ± 41.7; *p *< 0.001*) and fluoroscopy time (FP vs. RP: 20.9 min ± 10.6 vs 18.7 min ± 9.3; *p *= 0.035*) were lower in the RP cohort, although previous repositioning maneuvers were more common. Predilatation was less frequently observed in RP patients (FP vs. RP: 52.9% vs. 24.9%; *p *< 0.001*). Apart from vascular complications (FP vs. RP: 11.6% vs. 5.8%; *p *= 0.045*), all other intraprocedural complications were comparable. The average implantation depth (ID) was reduced in the RP cohort (FP vs. RP: − 5.5 mm ± 2.8 vs. − 4.9 mm ± 2.2; *p *= 0.023*), mostly driven by deep ID towards the left-coronary cusp (LCC).

### Detailed analysis of the implantation depth

As ID is known to depend on calcification burden and distribution, subgroups by the severity of aortic valve calcification (AVC), the left ventricular outflow tract (LVOT) calcification and morphology, and the aortic valve leaflet distribution were established, as well as a subdivision by chosen valve size. In brief, the highest average ID was reached under RP in severe AVC grading (FP vs. RP: − 5.3 mm ± 2.6 vs − 4.3 mm ± 2.1; *p *= 0.011*) and valve size 26 mm (FP vs. RP: − 4.9 mm ± 2.0 vs − 4.0 mm ± 1.9; *p *= 0.014*). For detailed information, please see Table 1.

**Table 1:** Impact on implantation depth (ID)

Characteristics	ID	FP (*n* = 189)	RP (*n* = 189)	*p* value
AVC grading				
Mild	ID→NCC	− 5.6 ± 3.4	− 4.5 ± 2.1	*0.031
	ID→LCC	− 7.0 ± 3.4	− 5.6 ± 2.1	*0.007
	Average ID	− 6.3 ± 3.3	− 5.1 ± 2.0	*0.011
Moderate	ID→NCC	− 4.5 ± 2.7	− 4.8 ± 2.5	0.525
	ID→LCC	− 6.0 ± 2.8	− 6.3 ± 2.4	0.686
	Average ID	− 5.3 ± 2.6	− 5.5 ± 2.3	0.583
Severe	ID→NCC	− 4.6 ± 2.9	− 3.7 ± 2.4	*0.036
	ID→LCC	− 6.1 ± 2.7	− 5.0 ± 2.4	*0.010
	Average ID	− 5.3 ± 2.6	− 4.3 ± 2.1	*0.011
LVOT-Calcification				
None	ID→NCC	− 5.2 ± 2.9	− 4.7 ± 2.1	0.190
	ID→LCC	− 7.0 ± 2.7	− 5.8 ± 2.2	*0.003
	Average ID	− 6.1 ± 2.7	− 5.3 ± 2.1	*0.003
Relevant	ID→NCC	− 4.1 ± 3.1	− 3.7 ± 2.4	0.114
	ID→LCC	− 5.6 ± 2.9	− 5.1 ± 2.3	0.175
	Average ID	− 5.0 ± 2.8	− 4.4 ± 2.2	0.111
Valve sizes				
23 mm	ID→NCC	− 4.3 ± 1.5	− 6.0 ± 2.0	0.315
	ID→LCC	− 4.0 ± 1.0	− 5.0 ± 1.7	0.435
	Average ID	− 4.2 ± 0.3	− 5.5 ± 1.8	0.275
26 mm	ID→NCC	− 4.4 ± 2.1	− 3.3 ± 2.1	*0.011
	ID→LCC	− 5.4 ± 2.3	− 4.6 ± 2.1	0.058
	Average ID	− 4.9 ± 2.0	− 4.0 ± 1.9	*0.014
29 mm	ID→NCC	− 5.0 ± 2.7	− 4.5 ± 2.2	0.198
	ID→LCC	− 6.5 ± 2.6	− 5.6 ± 2.1	*0.008
	Average ID	− 5.7 ± 2.4	− 5.1 ± 2.0	*0.036
34 mm	ID→NCC	− 4.9 ± 4.1	− 5.0 ± 2.6	0.977
	ID→LCC	− 6.8 ± 3.7	− 6.7 ± 2.7	0.843
	Average ID	− 5.9 ± 3.8	− 5.8 ± 2.5	0.934

Values are mean ± SD, median ± interquartile range or *n* (%)

*p-value<0.05

**Fig. 1:**
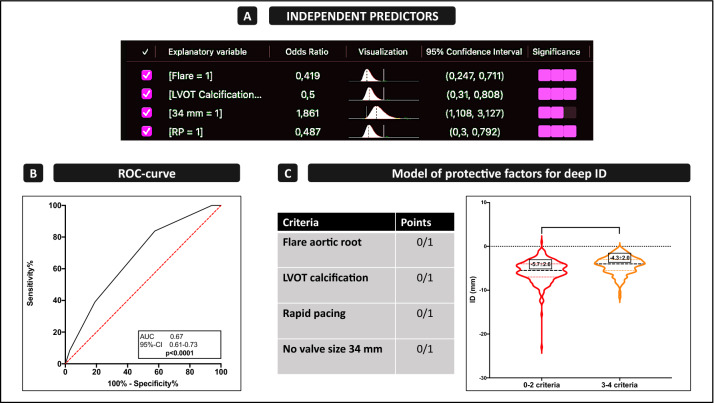
Independent predictors of very deep ID <  − 6 mm. **A** Identified predictors of a very deep implantation depth (ID) toward the LVOT. **B** C-statistics of the independent predictors. **C** Converted protective model that includes all independent predictors, resulting in a significantly higher ID depending on the number of criteria (0–2 criteria: − 5.7 mm ± 2.6 vs. 3–4 criteria − 4.3 mm ± 2.0; *p *< 0.0001****). *AUC* area under the curve

To establish univariate and multivariate binomial regression analysis, an average ID beneath − 6 mm as a dependent outcome variable for very deep implantation depth was selected according to the best practice recommendations. Covariates associated with deep ID <  − 6 mm in the univariate analysis (*p *< 0.1) were entered into the multivariate model (Table 2). The multivariate analysis identified LVOT calcification [OR 0.50 (0.31–0.81), *p *= 0.005*], a “flare” aortic root [OR 0.42 (0.25–0.71), *p *= 0.001*], and RP (OR 0.49 [0.30–0.79], *p *= 0.004*) as independent highly preventable predictors of a deep ID (Fig. 1A). Independent risk factors for deep ID were the use of a 34-mm device [OR 1.86 (1.11–3.13), *p *= 0.019*]. The C-statistic revealed a mediocre association of the aforementioned parameters combined with ID (Fig. 1B; Table 2: AUC = 0.67; 95% CI = 0.61–0.73; *p *< 0.0001).

**Table 2:** Univariate and multivariate regression analysis of average ID <  − 6 mm

	Univariate analysis		Multivariate analysis		ROC-curve (only independent predictors)		
	OR (95% CI)	*p* value	OR (95% CI)	*p* value	AUC	95% CI	*p* value
(A) Protective for deep ID							
Rapid pacing	0.57 (0.36–0.90)	0.016*	0.49 (0.30–0.79)	0.004*	0.67	0.61–0.73	< 0.0001*
LVOT Calcification	0.55 (0.35–0.87)	0.011*	0.50 (0.31–0.81)	0.005*			
“Flare” Aortic root	0.49 (0.29–0.81)	0.005*	0.42 (0.25–0.71)	0.001*			
Valve size 26 mm	0.51 (0.29–0.90)	0.020*	–	–			
(B) Risk for deep ID							
Annulus Perimeter	1.04 (1.01–1.07)	0.010*	–	–			
Annulus Diameter	1.14 (1.03–1.25)	0.010*	–	–			
LVOT Diameter	1.15 (1.05–1.26)	0.002*	–	–			
“Tube” Aortic root	2.00 (1.23–3.22)	0.005*	–	–			
Valve size 34 mm	2.02 (1.23–3.33)	0.006*	1.86 (1.11–3.13)	0.019*			

*p-value<0.05

According to the independent predictors, we built a model of protective criteria with integration of all of the aforementioned factors, which also included the risk factors as an inverted parameter, resulting in a distribution from zero to four criteria in summation of the several covariates. C-statistics according to Youden’s Index revealed that the critical cutoff for ID was > / < 2 points (sensitivity 84%, specificity 42%). Thus, ID was significantly reduced when comparing patients with two or less of the identified predictors and the summation of more than three of them (Fig. 1C; 0–2 criteria: − 5.7 mm ± 2.6 vs. 3–4 criteria − 4.3 mm ± 2.0; *p *< 0.0001*).

**Fig. 2:**
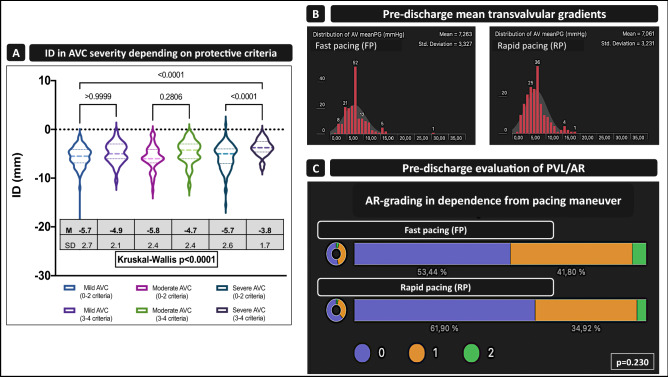
Thirty-day outcome and functional status. **A** Final ID according to different AVC severity and the applied criteria of the protective model. **B** Functional improvement—shown as the mean gradient (dPmean)—in the FP and RP cohorts. **C** Frequency distribution of paravalvular leakage-related aortic regurgitation (AR) comparing the FP and RP cohorts.

Although the severity of calcification failed to be a dependent or independent predictor, we discovered significant differences in ID as mentioned before and in Table 2 in dependency using different pacing maneuvers. Thus, ID was significantly higher in severe AVC when comparing patients with two or less of the identified predictors and the summation of more than three of them (Fig. 2A; 0–2 criteria: − 5.7 mm ± 2.6 vs. 3–4 criteria − 3.8 mm ± 1.7; *p *< 0.0001*), while no differences were seen comparing mild or moderate AVC.

### Thirty-day outcome and functional status

Whereas most of the outcome characteristics were comparable, FP patients accompanied showed a prolonged in-hospital and ICU stays (Supplementary material—Table 4), probably driven by more major vascular complications by trend. The need for permanent pacemaker implantation was similar in both cohorts.

Functional improvement was observed in both groups without differences concerning prosthesis function and paravalvular regurgitation as evaluated by the pre-discharge echocardiography (Fig. 2B, C).

## Discussion

Despite technological and procedural improvements in the past decade, TAVR is still associated with significantly higher PPI rates [13] primarily when using self-expanding devices [3, 4] Optimizing valve ID plays a crucial role in avoiding conduction disturbances and the need for PPI, and for achieving the best functional integrity of the new device. The impact of frequently used intraprocedural techniques, such as fast to rapid ventricular pacing maneuvers, on ID is still unclear.

The main read-outs of our retrospective study revealed the following:

1. Implantation depth was substantially reduced, with the highest ID under rapid pacing in severe aortic valve calcification and valve size 26 mm.
2. Rapid pacing was identified as a potent independent predictor for a higher ID.

### The choice of fast vs. rapid pacing

Several anatomical and technical considerations should be applied when selecting the best implantation strategy. Rapid pacing stabilizes the deployment by inhibiting cardiac output and it is essential for safe positioning of balloon expanding TAVR devices. Although it is less critical for repositionable, self-expanding devices, some criteria may influence towards an RP implantation strategy.

A potential risk of valve embolization can be identified by experienced implanters by taking into account left ventricular hypertrophy, calcification burden and distribution, shear forces caused by kinking or horizontal aorta, and hemodynamics. Especially in the sigmoid septal configuration, large anatomies, and mild calcification of the aortic valve, RP may be the better choice and is also recommended in clinical practice. In some cases, fast pacing alone cannot sufficiently reduce cardiac output at a stable level. However, some data report an exhausted right ventricular dysfunction during and after rapid pacing maneuvers [8]. In contrast, an already compromised hemodynamic profile, stable stroke volumes, and severe calcification in less complex anatomies are common reasons to maintain a fast or non-pacing strategy. Taking all of these considerations into account, it becomes urgent to elucidate why the baseline patient characteristics substantially differed concerning sex, age, risk scores, and calcification burden between the fast- and rapid-pacing cohorts in this non-selected TAVR all-comer cohort. Although one-to-one propensity match scoring was performed to balance this heterogeneity, some of the aforementioned characteristics still differed significantly including risk stratification and AVC. This phenomenon may be linked to the more frequently use of RP, starting with 6% (2015–2017) to nearly 75% in the last years at our center, also taken more experience, more treatment of mild AVC, and younger patients with a lower risk profile into account.

### Procedural characteristics

We observed several intraprocedural differences. First, predilatation maneuvers were less frequently used in RP patients, which may be caused by the selection bias of a milder AVC severity, as shown in the baseline characteristics. Furthermore, contrast medium use and fluoroscopic time were highly reduced in the RP cohort, although previous repositioning maneuvers were enhanced to reach an optimal ID. This phenomenon may be explained by dislocations with the need for a second valve implantation in the FP cohort, causing a need for more contrast agent during complex snaring procedures. However, with only three cases of dislocation, the statistically high reduction in contrast use cannot only be explained by these cases, but in turn by the higher number of vascular complications in the FP cohort.

### Impact on implantation depth

The average ID was significantly reduced in the RP cohort, mostly driven by deep and asymmetrical implantation towards the LCC. As ID is known to depend on anatomical criteria such as calcification burden and distribution, subgroups by AVC severity and leaflet calcification distribution, valve sizes, and LVOT calcification as well as morphology of the aortic root were established. The highest ID was reached under RP in severe AVC grading, a valve size of 26 mm, but also ID in mild AVC severity was optimized using rapid pacing maneuvers. Interestingly, ID was comparable in asymmetrical leaflet calcification and the largest self-expanding valve size, well-known factors leading to challenging positioning or possible valve dislocation: severe AVC, smaller devices and rapid pacing may allow more stable positioning, while mild AVC and large devices are known to be somewhat difficult to handle in terms of safe anchoring and dislocation movements.

To determine whether RP truly influences ID or is just a result of the heterogeneity in the different cohorts—although propensity matching was established—dependent and independent predictors of ID were calculated. Multivariate analysis depicted three protective (LVOT calcification, a flared configuration of the aortic root, and RP) and one risky covariate (valve size 34 mm) as independent predictors of a very deep implantation depth beneath − 6 mm toward the LVOT. In an established preventive model for very deep ID also including the inverted risk factor, the highest ID was reached in patients who had three to four of these independent predictors. Other aortic root parameters were only identified as dependent predictors.

LVOT calcification has been previously considered in the context of aortic regurgitation and conduction disturbances [14, 15] but with contrary trends. However, these studies only handled LVOT calcification as a risk factor for PPI need and not as a predictor for ID. LVOT calcification may inhibit micro- and macro-movements of the device toward the LVOT and, therefore, may enable a higher ID. The same may be applied to the role of a flared configuration of the aortic root, where the LVOT is smaller than the annulus. According to this, a more tubed configuration was identified as a dependent risk factor for a deep ID in this analysis but failed to be an independent predictor. Although AVC severity failed to be a predictor, we observed significant differences in ID using different pacing maneuvers. Thus, ID was significantly higher in severe AVC when comparing patients with two or less of the identified predictors and the summation of more than three of them, while no differences were seen in mild or moderate AVC, supposing that the severity of AVC plays a probably under-recognized role due to the propensity matching in this context. Until now, there have been no structured data about the role of RP on implantation depth aside from practical experience.

### Impact on 30 day outcomes

Whereas most of the outcome characteristics were comparable and device function showed favorable results in both cohorts, FP patients were documented with a significantly prolonged in-hospital and ICU stay. This phenomenon may be caused by the FP and RP cohorts’ different risk profiles, as mentioned above and in the baseline statistics. For example, the logistic EuroScore I was significantly lower in the RP cohort, suggesting a higher bleeding risk in the FP cohort. Also, major vascular complications were enhanced by trend in the FP cohort.

Unfortunately, the need for permanent pacemaker implantation was similar and high (approximately 15%) in both cohorts. The fact that—according to current knowledge—the targeted ID should be substantially higher may have contributed to this result. Atrioventricular block, atrial fibrillation, and a pre-existing right bundle branch block all increase the risk for pronounced conduction disturbances following TAVR [16]. Furthermore, the role of the membranous septum (MS) has become more evident in recent years. The MS is located inferior to the interleaflet triangle between the right and noncoronary aortic sinuses. It involves the atrioventricular (AV) node, which continues as the AV bundle of His through the MS lower border. A short MS and likewise deeper implantation depth are known to be associated with an increased risk for PPI [17]. Hence, Jilaihawi et al. developed an individualized approach for minimizing implantation depth according to the MS (MIDAS) [5]. According to this knowledge, the recommendations for best practice implantation of the Medtronic self-expanding device have changed from a target ID between − 3 and − 5 mm toward − 3 mm in the last year. Currently, a cusp overlap angulation technique is recommended to reach the highest ID as possible. Future studies have to clarify the impact of these recommendations on ID and the clinical outcomes in prospective cohorts.

## Conclusion

Data from this retrospective analysis indicate that rapid pacing is an independent predictor of reaching a higher implantation depth using self-expanding devices. Randomized studies should prove for validation compared to fast and non-pacing maneuvers during valve delivery and their impact on implantation depth.

### Limitations

This study is a single-center, retrospective analysis with associated unavoidable limitations due to its design. We did not account for the number of pacing episodes, supposing that only the kind of pacing during valve release—not during the initial positioning—influences the implantation depth. Implantation depth by angiography is notoriously tricky and may not correlate with MSCT depth assessment. Two experienced implanters provided commitment on the final ID intra-procedurally based on the angiographic results. Ideally, a correlation with MSCT to confirm the accuracy of the implant depth by angiography in a selected set of patients would strengthen the data; however, repeated MSCT without clinical indication is restricted by ethical governments in Germany.

## Supplementary Information

Below is the link to the electronic supplementary material.

Supplementary file1 (DOCX 26 KB)

## Data Availability

The datasets used and/or analyzed during the current study are available from the corresponding author on reasonable request.

## Abbreviations

ID: Implantation depth
LCC: Left coronary cusp
LVOT: Left ventricular outflow tract
NCC: Noncoronary cusp
MSCT: Multislice computer tomography
OR: Odds ratio
