# Supplementary material for: Factors associated with a high or low implantation of self-expanding devices in TAVR
Source: Clin Res Cardiol. 2021 Jun 24;110(12):1930–8. doi: 10.1007/s00392-021-01901-3 (PMC8639548; doi:10.1007/s00392-021-01901-3)
Supplement: Supplementary file 1 — Supplementary file1 (DOCX 26 KB) [file 392_2021_1901_MOESM1_ESM.docx]

**SUPPLEMENTARY MATERIAL**

**Table 1. Patient Clinical and Functional Characteristics**

| **Clinical data** | **Over-all**  **(n=473)** | **FP**  **(n=284)** | **RP**  **(n=189)** | **p-value** |
| --- | --- | --- | --- | --- |
| Age, years | 82.0 ± 5.4 | 82.3 ± 5.1 | 81.4 ± 5.8 | 0.078 |
| Gender, male | 221 (46.7) | 123 (43.3) | 98 (51.9) | 0.068 |
| BMI | 26.6 ± 4.8 | 26.3 ± 4.9 | 27.0 ± 4.7 | 0.093 |
| CAD | 345 (72.9) | 211 (74.3) | 134 (70.9) | 0.719 |
| Previous PCI | 182 (38.5) | 114 (40.1) | 68 (36.0) | 0.362 |
| Previous CABG | 40 (8.5) | 22 (7.8) | 18 (9.5) | 0.496 |
| Previous valve | 2 (0.4) | 0 (0.0) | 2 (1.1) | 0.082 |
| Previous PPI | 63 (13.3) | 39 (13.7) | 24 (12.7) | 0.746 |
| Arterial hypertension | 434 (91.8) | 265 (93.3) | 169 (89.4) | 0.132 |
| PHT | 306 (64.7) | 193 (68.0) | 113 (59.8) | 0.069 |
| Diabetes mellitus | 131 (27.7) | 78 (27.5) | 53 (28.0) | 0.747 |
| PAD | 104 (22.0) | 62 (21.8) | 42 (22.2) | 0.920 |
| CVD | 68 (14.4) | 35 (12.3) | 33 (17.5) | 0.119 |
| Previous RRT | 20 (4.2) | 12 (4.2) | 8 (4.2) | 0.997 |
| COPD | 133 (28.1) | 86 (30.3) | 47 (24.9) | 0.200 |
| **Functional data** |  |  |  |  |
| Log ES_I, % | 23.8 ± 14.7 | 25.4 ± 14.9 | 21.6 ± 14.0 | **0.004* |
| LVEF, % | 57.1 ± 20.8 | 57.3 ± 25.8 | 56.7 ± 9.0 | 0.753 |
| AVA, cm^2^ | 0.75 ± 0.2 | 0.73 ± 0.2 | 0.77 ± 0.2 | **0.012* |
| dPmax, mmHg | 62.0 ± 24.6 | 63.9 ± 24.6 | 50.0 ± 24.3 | **0.033* |
| dPmean, mmHg | 38.1 ± 16.7 | 39.6 ± 17.2 | 35.9 ± 15.6 | **0.020* |
| **MSCT data** |  |  |  |  |
| Annulus perimeter, mm | 76.5 ± 7.3 | 77.0 ± 7.5 | 75.8 ± 6.9 | 0.075 |
| Annulus mean diameter, mm | 24.4 ± 2.3 | 24.5 ± 2.4 | 24.1 ± 2.2 | 0.075 |
| LVOT mean diameter, mm | 23.4 ± 2.6 | 23.3 ± 2.6 | 24.5 ± 2.7 | 0.621 |
| Aortic root angulation, ° | 49.3 ± 8.9 | 49.5 ± 9.3 | 49.1 ± 8.2 | 0.604 |
| AVC, grading |  |  |  |  |
| mild | 129 (27.3) | 52 (18.3) | 77 (40.7) | **<0.001* |
| moderate | 106 (22.4) | 60 (21.1) | 46 (24.3) | 0.412 |
| severe | 238 (50.3) | 172 (60.6) | 66 (34.9) | **<0.001* |
| Asymmetrical Leaflet Calcification | 369 (78.0) | 226 (79.6) | 143 (75.7) | 0.314 |
| LVOT-Calcification | 237 (50.1) | 154 (54.2) | 83 (43.9) | **0.028* |
| Values are mean ± SD, median ± interquartile range or n (%).  AF=atrial fibrillation; AVA=aortic valve area; BMI=body mass index; CABG=coronary artery bypass graft; CAD=coronary artery disease; CI=cardiac index; COPD=chronic obstructive pulmonary disease; CVD=cerebrovascular disease; dPmean/max=mean/max. transvalvular gradient; LVEF=Left ventricular ejection fraction; LVOT=Left ventricular outflow tract; PCI=percutaneous coronary intervention; PHT=pulmonary hypertension; PAD=peripheral artery disease; PPI=permanent pacemaker implantation; RRT=renal replacement therapy; | | | | |

**Table 2. Patient Clinical and Functional Characteristics (propensity score matched analysis)**

| **Clinical data** | **Over-all**  **(n=378)** | **FP**  **(n=189)** | **RP**  **(n=189)** | **p-value** |
| --- | --- | --- | --- | --- |
| Age, years | 81.8 ± 5.5 | 82.2 ± 5.2 | 81.4 ± 5.8 | 0.171 |
| Gender, male | 185 (48.9) | 87 (46.0) | 91 (48.2) | 0.258 |
| BMI | 26.8 ± 4.8 | 26.5 ± 4.9 | 26.5 ± 4.9 | 0.309 |
| CAD | 275 (72.5) | 141 (74.6) | 134 (70.9) | 0.850 |
| Previous PCI | 145 (38.4) | 77 (40.7) | 68 (36.0) | 0.341 |
| Previous CABG | 35 (9.3) | 17 (9.0) | 18 (9.5) | 0.856 |
| Previous valve | 2 (0.5) | 0 (0.0) | 2 (1.1) | 0.156 |
| Previous PPI | 51 (13.5) | 27 (14.3) | 24 (12.7) | 0.652 |
| Arterial hypertension | 345 (91.3) | 176 (93.1) | 169 (89.4) | 0.202 |
| PHT | 248 (65.6) | 135 (71.4) | 113 (59.8) | **0.017* |
| Diabetes mellitus | 106 (28.0) | 53 (28.0) | 53 (28.0) | 1.000 |
| PAD | 85 (22.5) | 43 (22.3) | 42 (22.2) | 0.902 |
| CVD | 56 (14.8) | 23 (12.2) | 33 (17.5) | 0.148 |
| Previous RRT | 18 (4.8) | 10 (5.3) | 8 (4.2) | 0.629 |
| COPD | 111 (29.4) | 64 (33.9) | 47 (24.9) | 0.055 |
| **Functional data** |  |  |  |  |
| Log ES_I, % | 24.2 ± 15.1 | 26.9 ± 15.7 | 21.6 ± 14.0 | **<0.001* |
| LVEF, % | 57.0 ± 22.8 | 57.3 ± 31.0 | 56.7 ± 9.0 | 0.806 |
| AVA, cm^2^ | 0.77 ± 0.2 | 0.76 ± 0.2 | 0.77 ± 0.2 | 0.486 |
| dPmax, mmHg | 59.3 ± 23.7 | 59.5 ± 23.0 | 50.0 ± 24.3 | 0.856 |
| dPmean, mmHg | 36.6 ± 16.3 | 37.2 ± 17.0 | 35.9 ± 15.6 | 0.459 |
| **MSCT data** |  |  |  |  |
| Annulus perimeter, mm | 77.0 ± 7.4 | 78.2 ± 7.8 | 75.8 ± 6.9 | **0.002* |
| Annulus mean diameter, mm | 24.5 ± 2.7 | 24.9 ± 2.5 | 24.1 ± 2.2 | **0.002* |
| LVOT mean diameter, mm | 23.5 ± 2.7 | 23.5 ± 2.6 | 24.5 ± 2.7 | 0.735 |
| Aortic root angulation, ° | 49.5 ± 8.6 | 50.0 ± 9.0 | 49.1 ± 8.2 | 0.301 |
| AVC, grading |  |  |  |  |
| mild | 119 (31.5) | 42 (22.2) | 77 (40.7) | **<0.001* |
| moderate | 92 (24.3) | 46 (24.3) | 46 (24.3) | 1.000 |
| severe | 167 (44.2) | 101 (53.4) | 66 (34.9) | **<0.001* |
| Asymmetrical Leaflet Calcification | 285 (75.4) | 142 (75.1) | 143 (75.7) | 0.905 |
| LVOT-Calcification | 184 (48.7) | 101 (53.4) | 83 (43.9) | 0.064 |
| Values are mean ± SD, median ± interquartile range or n (%).  AF=atrial fibrillation; AVA=aortic valve area; BMI=body mass index; CABG=coronary artery bypass graft; CAD=coronary artery disease; CI=cardiac index; COPD=chronic obstructive pulmonary disease; CVD=cerebrovascular disease; dPmean/max=mean/max. transvalvular gradient; LVEF=Left ventricular ejection fraction; LVOT=Left ventricular outflow tract; PCI=percutaneous coronary intervention; PHT=pulmonary hypertension; PAD=peripheral artery disease; PPI=permanent pacemaker implantation; RRT=renal replacement therapy; | | | | |

**Table 3. Procedural Characteristics (propensity score matched cohort)**

| **Procedural data** | **Over-all**  **(n=378)** | **FP**  **(n=189)** | **RP**  **(n=189)** | **p-value** |
| --- | --- | --- | --- | --- |
| Valve sizes |  |  |  |  |
| 23 mm | 6 (1.6) | 3 (1.6) | 3 (1.6) | 1.000 |
| 26 mm | 101 (26.7) | 52 (27.5) | 49 (25.9) | 0.727 |
| 29 mm | 179 (47.4) | 80 (42.3) | 99 (52.4) | 0.050 |
| 34 mm | 92 (24.3) | 54 (28.6) | 38 (20.1) | 0.055 |
| Valve oversizing (%) | 97.1 ± 48.9 | 18.7 ± 8.1 | 20.8 ± 5.4 | **0.012* |
| CoreValve Evolut R^TM^_,_ | 360 (95.2) | 172 (91.0) | 188 (99.5) | **<0.001* |
| CoreValve EvolutPRO^TM^ | 18 (4.8) | 17 (9.0) | 1 (0.5) | **<0.001* |
| Contrast, ml | 97.1 ± 48.9 | 107.6 ± 53.3 | 86.6 ± 41.7 | **<0.001* |
| Fluoroscopy time, min | 19.8 ± 10.0 | 20.9 ± 10.6 | 18.7 ± 9.3 | **0.035* |
| Dose Area Product, Gy x cm^2^ | 3.947 ± 3.204 | 3.948 ± 3.187 | 3.946 ± 3.229 | 0.995 |
| Predilatation | 147 (38.9) | 100 (52.9) | 47 (24.9) | **<0.001* |
| Postdilatation | 40 (10.6) | 25 (13.2) | 15 (7.9) | 0.095 |
| Re-sheath/ -capture of valve | 59 (15.6) | 19 (10.1) | 40 (21.2) | **0.003* |
| **Functional data** |  |  |  |  |
| ARI | 24.2 ± 7.9 | 24.3 ± 8.3 | 24.1 ± 7.4 | 0.822 |
| AR > mild | 3 (0.8) | 3 (1.6) | 0 (0) | 0.082 |
| Implantation depth (NCC) | -4.5 ± 2.7 | -4.8 ± 3.0 | -4.3 ± 2.3 | 0.079 |
| Implantation depth (LCC) | -5.9 ± 2.6 | -6.3 ± 2.9 | -5.5 ± 2.3 | **0.008* |
| Average Implantation depth | -5.2 ± 2.5 | -5.5 ± 2.8 | -4.9 ± 2.2 | **0.023* |
| **Intraprocedural complications** |  |  |  |  |
| Immediate stroke | 1 (0.3) | 1 (0.5) | 0 (0) | 0.317 |
| Aortic dissection | 0 (0) | 0 (0) | 0 (0) | 1.000 |
| Annulus rupture | 0 (0) | 0 (0) | 0 (0) | 1.000 |
| Coronary obstruction | 0 (0) | 0 (0) | 0 (0) | 1.000 |
| Vascular complications | 33 (8.7) | 22 (11.6) | 11 (5.8) | **0.045* |
| Valve dislocation | 3 (0.8) | 3 (1.6) | 0 (0) | 0.082 |
| Conversion to surgery | 0 (0) | 0 (0) | 0 (0) | 1.000 |
| Need of 2nd valve | 3 (0.8) | 3 (1.6) | 0 (0) | 0.082 |
| Tamponade | 0 (0) | 0 (0) | 0 (0) | 1.000 |
| CPR | 2 (0.5) | 1 (0.5) | 1 (0.5) | 1.000 |
| Immediate Procedural death | 0 (0.0) | 0 (0.0) | 0 (0.0) | 1.000 |
| Disturbances of heart rhythm | 17 (4.5) | 9 (4.8) | 8 (4.2) | 0.805 |
| Values are mean ± SD, median ± interquartile range or n (%).  AR(I)=aortic regurgitation (index); CPR=cardiopulmonary resuscitation; | | | | |

**Table 4. Postprocedural outcome**

| **Postprocedural outcome** | **Over-all**  **(n=378)** | **FP**  **(n=189)** | **RP**  **(n=189)** | **p-value** |
| --- | --- | --- | --- | --- |
| 30-day mortality | 1 (0.3) | 1 (0.5) | 1 (0.0) | 0.317 |
| Disabling bleeding | 3 (0.8) | 2 (1.1) | 1 (0.5) | 0.562 |
| Major bleeding | 25 (6.6) | 16 (8.5) | 9 (4.8) | 0.147 |
| Major vascular complications | 25 (6.6) | 17 (9.0) | 8 (4.2) | 0.063 |
| Stroke | 10 (2.7) | 7 (3.7) | 3 (1.6) | 0.200 |
| AKI I-III | 50 (13.2) | 22 (11.6) | 28 (14.8) | 0.787 |
| New RRT | 9 (2.4) | 5 (2.7) | 4 (2.1) | 0.736 |
| New AVB (I-III°) | 59 (15.6) | 30 (15.9) | 29 (15.3) | 0.887 |
| New LBBB/RBBB | 47 (12.4) | 27 (14.3) | 20 (10.6) | 0.275 |
| New PPI | 61 (16.1) | 33 (17.5) | 28 (14.8) | 0.485 |
| In-hospital stay, days | 11.0 ± 7.1 | 13.2 ± 7.6 | 8.9 ± 5.8 | **<0.001* |
| ICU stay, days | 2.4 ± 3.1 | 3.1 ± 3.1 | 1.7 ± 2.9 | **<0.001* |
| Values are mean ± SD, median ± interquartile range or n (%).  AKI=acute kidney injury; AR=aortic regurgitation; AVB=atrioventricular block; ICU=intensive care unit; LBBB=left-bundle branch block; PPI=permanent pacemaker therapy; RBBB=right-bundle branch block; RRT=renal replacement therapy; | | | | |
